# ﻿*Cyrtomiumadenotrichum* (Dryopteridaceae), a new species from Guangxi, China

**DOI:** 10.3897/phytokeys.243.127579

**Published:** 2024-06-25

**Authors:** You Nong, Li-Qun Lei, Zi-Yi Zhao, Gui-Yuan Wei, Chuan-Gui Xu, Bin Feng, Xin-Cheng Qu, Ri-Hong Jiang

**Affiliations:** 1 Guangxi Key Laboratory of Traditional Chinese Medicine Quality Standards, Guangxi Institute of Chinese Medicine & Pharmaceutical Science, No. 20–1 Dongge Road, Nanning, Guangxi, China Guangxi Key Laboratory of Traditional Chinese Medicine Quality Standards, Guangxi Institute of Chinese Medicine & Pharmaceutical Science Nanning China; 2 Nanning Botanical Garden, Nanning Qingxiushan Scenic and Historic Tourism De-velopment Co.,Ltd, Nanning, Guangxi, China Nanning Botanical Garden, Nanning Qingxiushan Scenic and Historic Tourism De-velopment Co.,Ltd Nanning China; 3 Guangxi Key Laboratory of Special Non-wood Forest Cultivation and Utilization, Guangxi Forestry Research Institute, Nanning, 530002, China Guangxi Key Laboratory of Special Non-wood Forest Cultivation and Utilization, Guangxi Forestry Research Institute Nanning China

**Keywords:** Gully, limestone, Nandan, new species, taxonomy

## Abstract

*Cyrtomiumadenotrichum* Y. Nong & R.H. Jiang (Dryopteridaceae), a new species from Guangxi, China, is described and illustrated. This new species is similar to *C.nephrolepioides* (Christ) Copel., *C.obliquum* Ching & K. H. Shing ex K. H. Shing, *C.sinningense* Ching & K. H. Shing ex K. H. Shing and *C.calcis* Liang Zhang, N.T.Lu & Li Bing Zhang in having erect rhizomes, dense, leathery lamina and rounded sori, but it can be easily distinguishable by its stipe sparsely glandular, base obvious oblique, basiscopic base truncate, acroscopic base auriculate or ovate.

## ﻿Introduction

*Cyrtomium* (Presl 1836) was founded upon the basis of *Polypodiumfalcatum* (Linnaeus 1781), originating from Japan. It comprises approximately 40 recognized species, the majority of which are found in East Asia, with a particular concentration of diversity centered in Southwest China. Within this group, 31 species are native to China (Zhang and Barrington 2013). Cyrtomiumser.Falcata Ching & Shing (Shing 1965), which was not confirmed as monophyletic in an earlier molecular study conducted (Lu et al. 2005), is distinguished by its leathery leaves and pinnae, which possess intact (occasionally repand) and thickened margins. Notably, all species belonging to this series can be located in China, except for *C.elongatum* S.K.Wu & P.K.Lôc (Wu et al 2005) and most of them are naturally distributed in limestone regions. Within the past decade, more new species of *Cyrtomium* have been discovered in Vietnam and China (Lu et al 2023; Nong et al. 2023).

During our field surveys conducted in Nandan County, Guangxi, in March 2024, we encountered a unique population of *Cyrtomium* that exhibited morphological similarities to the species *C.nephrolepioides* (Christ) Copel.(Copeland 1929), *C.obliquum* Ching & K. H. Shing ex K. H. Shing (Shing 1965), *C.sinningense* Ching & K. H. Shing ex K. H. Shing (Shing 1965) and *C.calcis* Liang Zhang, N.T.Lu & Li Bing Zhang (Lu et al 2023) in having erect rhizomes, dense, leathery lamina, and rounded sori, but it can be easily distinguished by its stipe sparsely glandular, base obvious oblique, basiscopic base truncate, acroscopic base auriculate or ovate. We hypothesize that this unique population may represent a previously unrecognized species due to these distinct morphological characteristics. To further validate our findings, we conducted additional observations and examined numerous specimens of *Cyrtomium* housed in various herbaria. We also consulted relevant literature to ensure the accuracy of our identification and to gain a deeper understanding of the taxonomic status of this potential new species (Lu et al 2023; Nong et al. 2023). We describe this population as a new morphologically distinct species.

## ﻿Materials and methods

The new species was described based on field observations made in March and examination of herbarium specimens at GXMI. Other related *Cyrtomium* species were examined based on online images from Kew Herbarium Catalogue (http://apps.kew.org/herbcat/gotoHomePage.do) and JSTOR Global Plants (http://plants.jstor.org/) and PE, IBK and KUN. Morphological characters that distinguish it from all other species in the genus of *Cyrtomium* are used. We also observed living plants of the new species. We observed characters of rhizome, leaves, pedicels, stipe, lamina, scales, sori, indusia.

Descriptions were written from herbarium specimens. Measurements were made with a tape–measure and callipers. The structure of the indumentum and its distribution was observed and described under a dissecting microscope at magnifications of more than 20×. Additional information on locality, habitat, ecology, plant form and fruits were collected in the field and taken from herbarium labels. Conservation threat assessment followed IUCN Categories and Criteria (IUCN 2022).

## ﻿Results and discussion

### ﻿Taxonomy

#### ﻿ Cyrtomium adenotrichum

Taxon classificationPlantaePolypodialesDryopteridaceae

Y.Nong & R.H.Jiang sp. nov.

3B59821A-2F1F-5B8F-B8B7-4F04C25EB799

urn:lsid:ipni.org:names:77344264-1

Figs 1 , 2 , 3 , 4

##### Diagnosis.

*Cyrtomiumadenotrichum* is similar to *C.nephrolepioides*, *C.obliquum*, *C.sinningense* and *C.calcis*, but differs in its stipe sparsely glandular (vs. glabrous). In addition, it can be distinguished from *C.sinningense* by its scale margins fimbriate (vs. dentate), lateral pinnae 5–10 pairs (vs. 1–4 pairs), indusia margins dentate (vs. subentire); it can also be distinguished from *C.nephrolepioides* by its lateral pinnae 5–10 pairs (vs. 10–26 pairs), base obvious oblique (vs. cordate or sometimes obliquely cordate). It differs from *C.obliquum* by its scale margins fimbriate (vs. dentate), lateral pinnae 5–10 pairs (vs. 12–21 pairs), indusia margins dentate (vs. entire). It can be distinguished from *C.calcis* by its base obvious oblique (vs. cordate to hastate), lateral pinnae thin leathery (vs. thick leathery). Comparative morphological differences among all five species are presented in Table 1.

**Table 1.** Main morphological differences amongst *Cyrtomiumadenotrichum* and *C.nephrolepioides*, *C.obliquum*, *C.sinningense* and *C.calcis*.

Morphological traits	*C.adenotrichum*	*C.nephrolepioides*	*C.obliquum*	*C.sinningense*	*C.calcis*
Plant height	5–15 cm	12–28 cm	20–35 cm	8–12 cm	13–23 cm
Stipe	3–10 cm, 1 mm in diam., sparsely glandular	3–10 cm, 1–2 mm in diam., glabrous	6–10 cm, 1–2 mm in diam., glabrous	5–7 cm, 1 mm in diam., glabrous	15 cm, 1–3 mm in diam., glabrous
Scales margins	fimbriate	fimbriate	dentate	Dentate	fimbriate-dentate
Lamina	linear-lanceolate, 5–10 × 1.5–2 cm	linear-lanceolate, 10–25 × 2–5 cm	lanceolate, 13–35 × 3–5 cm	ovate or oblong-lanceolate, 3–7 × 2.5–3 cm	lanceolate-oblong, 13–23 × 1.9 cm
Lateral pinnae	5–10 pairs	10–26 pairs	12–21 pairs	1–4 pairs	9–14 pairs
Pinnae	0.8–1 × 0.4–0.6 cm	1–2.5 × 0.6–1.2 cm	2–3 × 1–1.5 cm	1.2–1.6 × 1–1.2 cm	1.5–3.5 × 1.2–1.9 cm
Base	obvious oblique	cordate or sometimes obliquely cordate	oblique	broadly cuneate	cordate to hastate
Texture	thin leathery	thick- leathery	leathery	Leathery	thick leathery
Venation	midrib flat or slightly concave on both surfaces	midrib concave on both surfaces	slightly raised abaxially, slightly concave adaxially	indistinct on both surfaces	obscure
Rows of areolae	1 or 2	2 or 3	2	2 or 3	2, 3, rarely to 4
Indusia margins	dentate	subentire	entire	subentire	dentate

**Figure 1.**
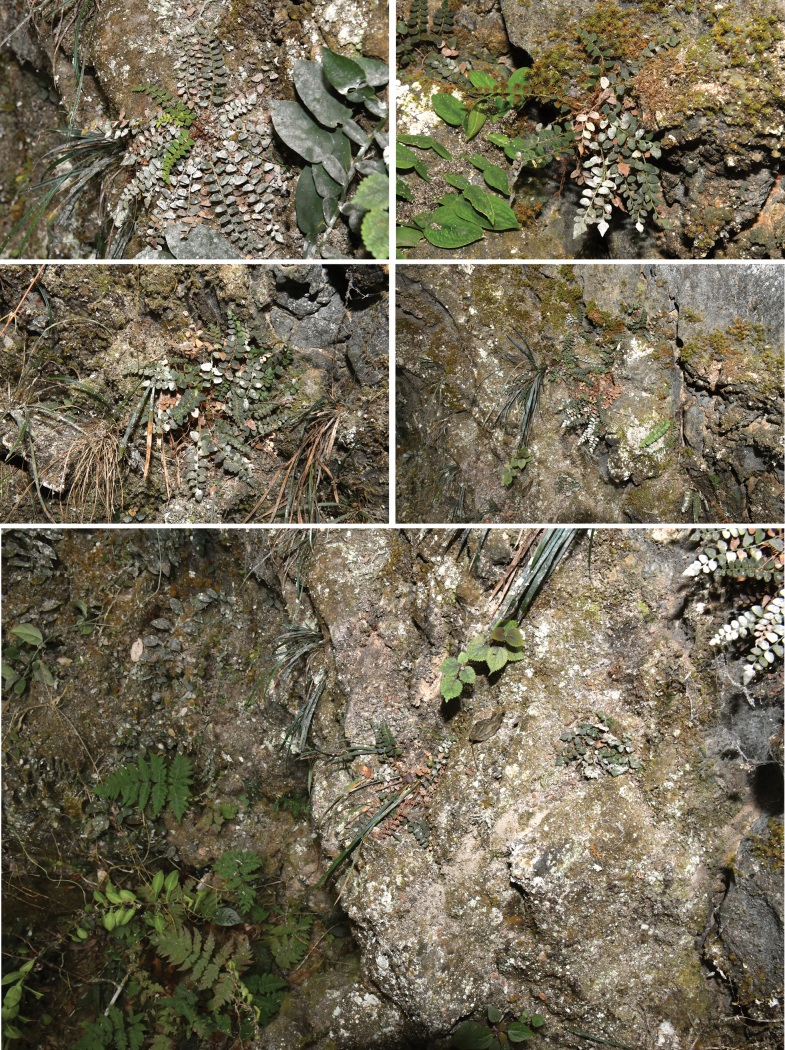
Habitat of *Cyrtomiumadenotrichum* Y. Nong & R.H. Jiang on cliffs at a gully (Photographed by YN).

##### Holotype.

China. Guangxi: Nandan, 24°48'47"N, 107°27'12"E, alt. 470 m, on the cliff at a gully; 17 March 2024; *Y Nong NY2024031701* (GXMI!). (holotype: GXMI!; isotypes: IBK!).

**Figure 2.**
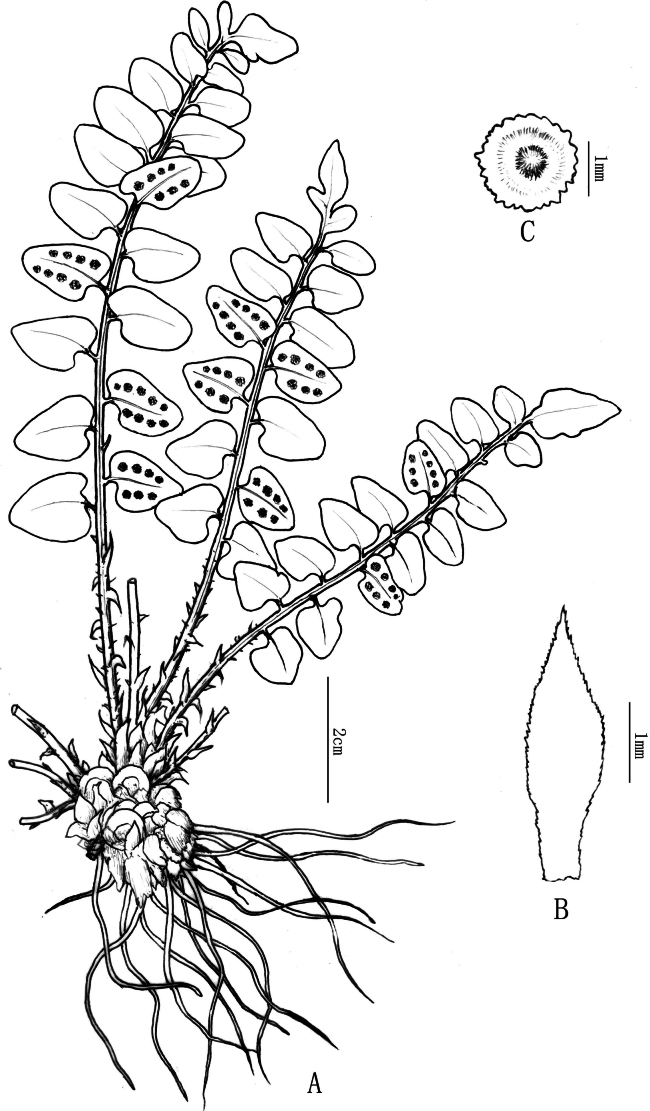
Line drawing of *Cyrtomiumadenotrichum* Y. Nong & R.H. Jiang **A** plant **B** scale **C** indusium (Drawn by Xin–Cheng Qu).

##### Description.

Plants perennial, evergreen, 5–15 cm tall. Rhizome short and erect, together with basal stipe densely scaly. Scales brown, ovate; Leaves clustered, petiole 1–3 cm, stipe stramineous, 3–10 cm, 1 mm in diam, sparsely glandular, densely scaly; scales brown, ovate or lanceolate, margins fimbriate. scales on stipe base brown, ovate or lanceolate, membranous, ca. 8–12 × 1–3 mm, margin minutely denticulate and slightly long ciliate, upword gradually narrowed, subulate, linear-lanceolate. Lamina linear-lanceolate, 5–10 × 1.5–2 cm, base not contracted, 1-imparipinnate; Lateral pinnae 5–10 pairs, crowded, alternate, spreading or slightly ascendant, shortly stalked, ovate or rarely deltoid-lanceolate; lower and middle pinnae 8–10 × 4–6 mm, respectively, subopposite or alternate, apex rounded, base obvious oblique, basiscopic base truncate, acroscopic base auriculate or ovate, margins entire and often slightly reflexed, sparse hairlike scales adaxially and abaxially; terminal pinna ovate, with 1 or 2 connate lobes at base, 20–35 × 15–25 mm; rachis c. 1 mm in diam, sparsely glandular, grooved adaxially, scaly abaxially; scales on rachis brown, linear to subulate, margins sparsely toothed or fimbriate; frond texture thin leathery; venation pinnate, midrib flat or slightly concave on abaxially and adaxially, lateral vein connection, indistinct, lateral veins anastomosing to form 1 or 2 rows of areoles on each side of midrib. Sori 1 row on each side of midrib; indusia margins dentate.

**Figure 3.**
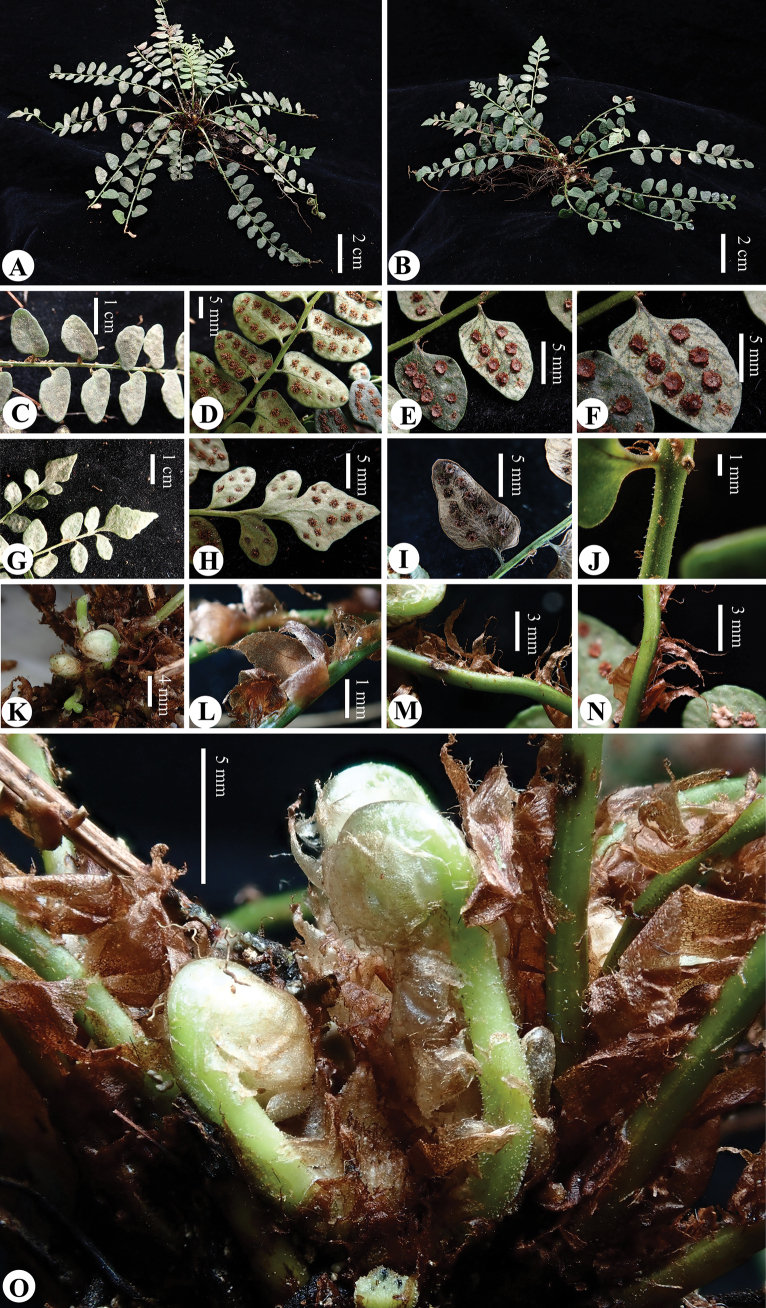
*Cyrtomiumadenotrichum* Y. Nong & R.H. Jiang **A, B** plant **C, D** lamina (adaxially and abaxially view) **E, F** sori and indusia **G, H** terminal pinna (adaxially and abaxially view) **I** lateral pinnae (abaxially view, showing: margins entire and often slightly reflexed) **J** stipe (sparsely glandular) **K** curled leaves **L, M, N** scales **O** curled leaves (sparsely glandular) (Photographed and edited by You Nong).

##### Etymology.

The specific epithet refers to the stipe sparsely glandular of the new species.

**Figure 4.**
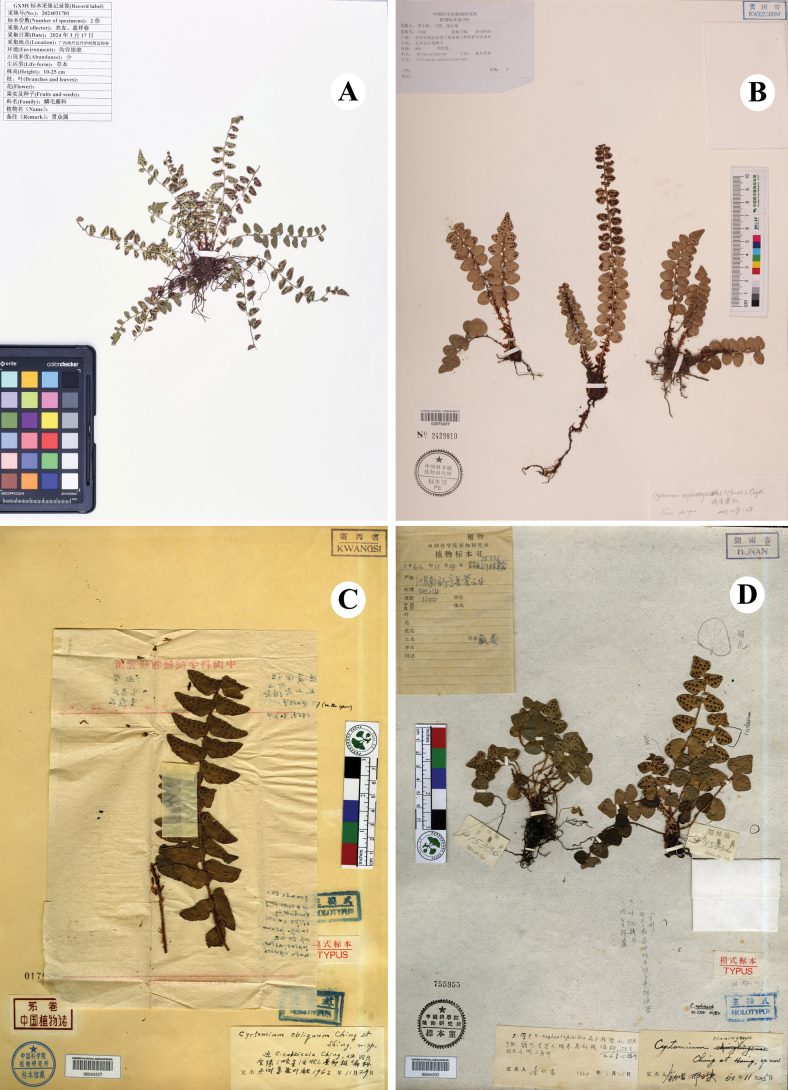
*Cyrtomium* specimens of the new taxon and three morphologically related species **A** type specimen of *Cyrtomiumadenotrichum***B***C.nephrolepioides***C** type specimen of *C.obliquum*, and **D** type specimen of *C.sinningense*.

##### Distribution and habit.

**Figure 5.**
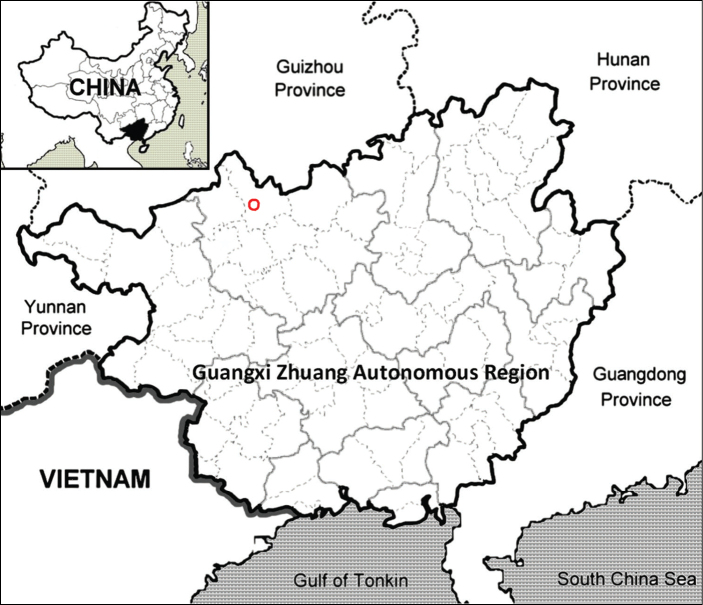
The distribution of *Cyrtomiumadenotrichum* (red circle) in Guangxi, China.

Known only from the north of Guangxi, China (Fig. 5). It has been mainly found on cliffs at a gully at elevations of 470 m. We found only one population with 10 individuals, and the habitat of *Cyrtomiumadenotrichum* is fragile because it could be submerged during the rainy season.

##### IUCN Red List Category.

Only one population with 10 individuals of *Cyrtomiumadenotrichum* is currently known from Nandan County, Guangxi China. Due to its rarity, the low number of individuals and habitat vulnerability, *C.adenotrichum* is considered to be Critically Endangered (CR), according to the IUCN (IUCN 2022).

## Supplementary Material

XML Treatment for Cyrtomium adenotrichum

## ﻿Additional information

### Author ORCIDs

You Nong https://orcid.org/0000-0001-7004-0946

Li-Qun Lei https://orcid.org/0009-0009-7319-5439

Zi-Yi Zhao https://orcid.org/0000-0003-2513-0728

Gui-Yuan Wei https://orcid.org/0000-0003-0652-1213

Chuan-Gui Xu https://orcid.org/0009-0000-6263-3821

Bin Feng https://orcid.org/0000-0003-0387-3635

Xin-Cheng Qu https://orcid.org/0009-0009-9078-9976

Ri-Hong Jiang https://orcid.org/0000-0003-4908-3487

### Data availability

All of the data that support the findings of this study are available in the main text.
